# The Use of Artificial Intelligence in Planning Dental Implant Procedures: A Systematic Review

**DOI:** 10.3390/dj14050248

**Published:** 2026-04-23

**Authors:** Gulvash Zaman, Rabia S. Khan, Adam Spacey, Cemal Ucer, Simon Wright

**Affiliations:** 1 ICE Postgraduate Dental Institute and Hospital, 24 Furness Quay, Salford M50 3XZ, UK; gulvash_zaman@hotmail.com (G.Z.); ucer@icedental.institute (C.U.); profwright@glencairndental.co.uk (S.W.); 2 School of Health and Society, University of Salford, Salford M5 4WT, UK; a.spacey@salford.ac.uk

**Keywords:** artificial intelligence, implant dentistry, accuracy, implant success, cone beam computed tomography, AI-assisted planning

## Abstract

**Background:** Artificial intelligence (AI) is increasingly being integrated into dental implantology, particularly in treatment planning, a critical phase for implant success. Traditionally dependent on clinician expertise, planning can now be supported by AI-assisted systems that aim to improve diagnostic accuracy, precision, and efficiency. Objective: To synthesise recent evidence on the use of AI in dental implant planning, particularly its ability to analyse cone beam computed tomography (CBCT) imaging to identify edentulous regions and assess bone dimensions compared with conventional planning methods. **Methods:** A systematic search was conducted across PubMed, Scopus, Google Scholar, and the Cochrane Library, with additional manual searches from October 2024 to July 2025. Eligibility was defined using the Population, Intervention, Comparison, Outcome (PICO) framework, focusing on adults undergoing implant procedures planned using AI-assisted CBCT imaging and deep learning (DL) models, particularly U-Net architectures, for CBCT segmentation. **Results:** Ten studies were included, AI systems demonstrated high accuracy (92–99.7%) in detecting teeth and edentulous regions, with precision and recall frequently exceeding 90%. AI-assisted planning also showed improved efficiency, and, in one study, higher implant success rates compared with traditional planning (92% vs. 78%). However, variability in study design, inconsistent reporting, and limited ethical oversight were noted. **Conclusions:** AI, particularly DL models applied to CBCT imaging, shows strong potential to enhance diagnostic precision and efficiency in dental implant planning. Nevertheless, the field requires standardised evaluation metrics, larger datasets, and well-designed clinical trials before widespread clinical implementation.

## 1. Introduction

Dental implants have become a cornerstone of modern restorative dentistry, providing a fixed, reliable, and long-lasting solution for tooth replacement. Unlike conventional prosthetics, implants preserve jawbone structure, maintain adjacent tooth integrity, and significantly improve oral function and aesthetics, resulting in enhanced patient quality of life [1]. Their success has been well-documented across both partially dentate and fully edentulous patients, with studies reporting high survival rates and favourable long-term outcomes [2]. However, these outcomes are not guaranteed and depend heavily on precise, well-executed treatment planning [3,4]. Effective implant planning involves careful evaluation of anatomical structures, occlusion, and patient-specific factors such as bone volume, density, and overall oral health. It plays a critical role in ensuring accurate implant positioning, minimizing complications, and improving both surgical and prosthetic outcomes [5]. Radiographic assessment, particularly through the use of cone beam computed tomography (CBCT), has become an essential diagnostic tool in this process, offering detailed three-dimensional imaging of bone morphology, nerve pathways, and sinus cavities [6,7]. When combined with computer-aided design and manufacturing (CAD/CAM), clinicians are able to fabricate surgical guides that improve precision and predictability in implant placement [8,9]. More recently, the incorporation of AI into clinical workflows has begun to significantly transform the methodology and precision of dental implant treatment planning. AI, particularly through deep learning (DL) techniques such as convolutional neural networks (CNNs), enables automated interpretation of CBCT scans, identifying edentulous regions, assessing bone dimensions, and aiding in virtual implant placement with a high degree of accuracy [10,11]. These systems can enhance clinical efficiency, reduce variability caused by human interpretation, and support better surgical outcomes.

While digital tools like CBCT and intraoral scanners have already improved the technical workflow, AI introduces a new level of diagnostic precision and decision-making support [12]. This is especially relevant given the inherent subjectivity and potential for human error in clinical judgement. AI systems, when properly trained, can support clinicians by providing consistent, data-driven analyses, thus reducing risks associated with anatomical misinterpretation or planning errors.

Despite its promising capabilities, the application of AI in dental implantology remains relatively novel. The existing literature often focuses on diagnostic applications, with fewer studies evaluating its role in treatment planning itself. A recent study by Alqutaibi et al. [13] examined AI’s ability to detect missing teeth and assess bone dimensions from CBCT scans, suggesting strong potential for improving planning accuracy. However, the evidence base is still emerging, and more comprehensive evaluations are needed to understand AI’s full impact in clinical settings.

This review aims to fill that gap by critically examining current research on AI-assisted implant planning, particularly in the context of CBCT image analysis. By evaluating the effectiveness of AI technologies in identifying implant sites, measuring bone, and supporting treatment workflows, this study seeks to clarify AI’s role in enhancing implant success, surgical precision, and overall patient outcomes.

## 2. Materials and Methods

### 2.1. Review Protocol and Reporting Standards

This systematic review was conducted in accordance with Preferred Reporting Items for Systematic Reviews and Meta-Analyses (PRISMA) guidelines [14].

### 2.2. Eligibility Criteria

The eligibility criteria for study selection were based on the PICO (Population, Intervention, Comparison, Outcome) framework [15], ensuring a structured and focused review process Table 1.

To ensure the review’s clinical relevance, systematic reviews, case reports, expert opinions, in vitro studies, finite element analysis studies, and abstract-only publications were excluded. These study types were excluded due to a lack of clinical outcome data or low levels of evidence [17].

### 2.3. Search Strategy

A comprehensive literature search was conducted in October 2024, with a final search performed in July 2025 (from 2014–2025) to capture the most recent publications. The search covered five major electronic databases: PubMed/MEDLINE, Scopus, Google Scholar, and the Cochrane Library.

To maximise the sensitivity and relevance of the search, a combination of keywords, Medical Subject Headings (MeSH) terms, and Boolean operators was used. The following terms were combined:

(“Dental implant” OR “dental implant success” OR “dental implant outcome” OR “dental implant aesthetics” OR “dental implant precision” OR “dental implant planning”) AND (“Artificial intelligence” OR “AI” OR “machine learning” OR “deep learning”).

Additional manual searches were carried out via: The British Dental Journal (BDJ), Reference lists of relevant studies, and grey literature databases.

Search results were imported into Mendeley reference manager, which was used to organise studies and remove duplicates. The remaining records were screened in two stages: (1) titles and abstracts were reviewed against the inclusion criteria; (2) full texts were examined to confirm alignment with the study objectives and eligibility criteria.

### 2.4. Data Extraction and Study Characteristics

Key information from eligible studies was extracted and summarised in a structured Study Characteristics Table (Table 1). The data included:

• Authors and year of publication
• Study design
• Country and institution of origin
• Type and size of dataset
• Data collection timeframe
• Area of interest (e.g., detection of edentulous areas, bone dimension analysis)
• Stated objectives of each study

This standardised format facilitated consistent comparison and synthesis of the included literature. Data extraction was initially carried out and the following study characteristics and outcome measures were extracted to allow comparison between AI-assisted and conventional implant planning methods: study design, sample size, AI model or algorithm used, CBCT imaging parameters, accuracy of edentulous region detection, bone dimension assessment, diagnostic precision, sensitivity and specificity where reported, and reported clinical outcomes such as implant placement accuracy or success rate. These characteristics were summarised and compared across studies, as presented in Table 2.

### 2.5. Quality Assessment

The methodological quality of all included studies was assessed using the Critical Appraisal Skills Programme (CASP) checklists, which provide structured appraisal tools for a variety of study designs, including randomised controlled trials (RCTs), observational studies, and cohort studies.

The CASP tool guided evaluation in key areas, such as:

Clarity of research aims
Appropriateness of study design
Transparency of data collection methods
Consideration of ethical issues
Rigour of data analysis

Each study was reviewed over the course and a Quality Assessment Table (Supplementary Materials Table S1) summarises the results, with “Can’t tell” used when full-text papers lacked sufficient detail.

Although variability was noted in areas such as sample size justification and ethical reporting, all studies were considered to meet the minimum quality threshold for inclusion. Full CASP checklists for each study are available in Supplementary Materials Table S2.

### 2.6. Data Synthesis

A narrative approach was employed for data synthesis in this systematic review. The data synthesis process was conducted. Quantitative findings from the studies included were summarized descriptively, with key statistical outcomes highlighted where available. Integration of the data was guided by a convergent synthesis design, in which findings were initially analysed separately and subsequently brought together to inform the overall conclusions. The synthesis and interpretation of the results were cross-checked with any discrepancies resolved through discussion.

## 3. Results

Following a comprehensive electronic search across five major databases, a total of 3199 articles were initially identified. After the removal of 1845 duplicates, 1354 records remained for title and abstract screening. These were assessed against the predefined inclusion and exclusion criteria, leading to the exclusion of 1275 articles that did not meet the eligibility requirements. A further screening of the remaining 79 studies was cconducted, and 16 articles were deemed potentially relevant and selected for full-text review. Of these, nine studies were excluded due to not meeting the inclusion criteria. One study was excluded because it was conducted in vitro and did not involve real patient data [18]. Several others were excluded for relying on two-dimensional imaging methods, such as panoramic or periapical radiographs, rather than CBCT, which was required for inclusion [18,19,20,21]. Additionally, two studies focused primarily on the identification or classification of dental implant systems using AI, rather than on planning potential implant workflows, which fell outside the scope of this review [22,23]. One study was excluded due to its focus on the restoration phase of a dental implant [24]. Finally, two studies were excluded for lacking relevance to AI entirely, focusing instead on robotics and digital dentistry applications [25,26].

Furthermore, manual citation searching identified seven further studies for potential inclusion. After full-text assessment, four of these were excluded for reasons consistent with those applied during the database screening. One study was excluded due to the use of 2D imaging [26]. Another was excluded as it focused on the detection of an implant system using AI [27], and the remaining two studies were excluded because they were unrelated to the review’s scope, with one addressing digital workflows and the other investigating peri-implant bone loss [28,29]. Following both the electronic and manual search processes, a total of ten studies met the eligibility criteria and were included in this review.

**Figure 1:**
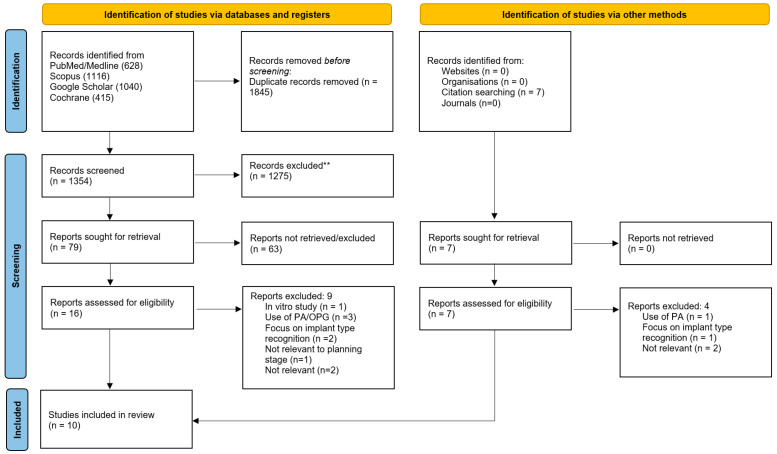
PRISMA flowchart representing the study selection and screening process.

Figure 1 is a PRISMA flowchart representing the study selection and screening process. This systematic review was conducted in accordance with the Preferred Reporting Items for Systematic Reviews and Meta-Analyses (PRISMA) 2020 guidelines. The PRISMA flow diagram describing the study selection process is presented in Figure 1, and the completed PRISMA checklist is provided as Supplementary Materials Table S1. The 10 studies’ characteristics included in this review are presented in chronological order in Table 2, and the AI technology used and outcomes of the study are presented in the Section 3 in Table 4.

**Table 2:** Study characteristics table.

Authors + Year	Study Type	Institution/Country	Dataset Collection Point	Dataset Size and Image Type	Area of Interest	Aim of Study
Bayrakdar et al., 2021 [30]	Non- interve ntional retrosp ective	Faculty of Dentistry of Eskişehir Osmangazi University, Turkey	2019	75 CBCTs	Alveolar bone and missing tooth regions	Assess reliability of AI in locating missing teeth regions, anatomical structures and calculating bone length/width in those areas.
Gerhardt et al., 2022 [31]	Observational	Centre of Dentomaxillofacial Radiology of the University Hospitals, Belgium	March 2016–January 2021	46 CBCTs Used after clinical validation. (23 full Dentate + 23 partially) (before there were 175–140 training, 35 testing)	Teeth and small edentulous regions	Primary outcome: to detect and classify teeth and missing teeth. Secondary outcome: time analysis and accuracy of tooth segmentation.
Al-Sarem et al., 2022 [32]	Non- interve ntional retrosp ective	Taibah University Dental Hospital, Saudia Arabia	2018–2022	500 CBCTs (Training 70%, validation 20%, testing 10%)	Areas of missing teeth	To enhance 3D missing tooth area detection for implant planning
Bodhe et al., 2022 [33]	Non-interventional retrospective	Maxwell Dental Clinic and Dr. Chandhok’s Multi-Speciality Dental Clinic, India	N/A	500 patients, 800 CBCTs. (Training = 80%, validation and testing = 20%).	Areas of missing teeth And implant indication. (Partially dentate or edentulous)	To test AI performance in implant planning, design, missing teeth detection and success prediction.
Moufti et al., 2023 [34]	Retrospective	University dental hospital, Sharjah, United Arab Emirates	N/A	43 CBCTs: 33 Training and 10 testing	Areas missing lower teeth	To identify areas of missing teeth for dental implant treatment, and to delineate the bone available.
Rajan et al., 2024 [35]	Randomised control trial	N/A	N/A	N/A	Areas of missing teeth	To evaluate the role of AI in predicting success of implant based on pre-op CBCTs
Satapathy et al., 2024 [36]	Observational	N/A	N/A	20 CBCTs	Areas of missing teeth needing implants	To investigate the use of AI- assisted treatment planning by comparing AI- generated plans with traditional clinical plans.
Al-Asali et al., 2024 [37]	Non- interve ntional retrosp ective	Taibah University Dental Hospital, Saudi Arabia	2018–2023	150 CBCTs	Area of missing teeth in patients aged 16–72	To enhance the efficiency and precision of dental implant placement by accurately determining the position of missing teeth by segmenting CBCT scans using AI.
Elgarba et al., 2024 [38]	Retrospective	UZ Leuven Hospital, Belgium	N/A	10 CBCTs and intraoral scan: 360 observations.	Areas of single missing teeth in the mandibular molar or premolar region.	To validate the quality and clinical acceptance of an AI tool for implant placement in single mandibular edentulous premolar/molar regions by comparing its performance with a human expert.
Alotaibi et al., 2025 [39]	Non- interve ntional retrosp ective	King Saud University, College of Dentistry	N/A	530 CBCT	To identify propose d implant position	Planning for implant size and position to classify different intra-osseous lengths (6, 8, 10) and different intraosseous diameters (3.3, 4.10)

Table 2, shows a total of ten studies were included in this systematic review, comprising a mix of retrospective studies, observational studies and a randomised controlled trial. The studies were conducted across diverse geographic regions, including Turkey, Belgium, Saudi Arabia, India, the United Arab Emirates, and potentially others, reflecting a growing global interest in the application of AI in dental implantology. All included studies used CBCT scans as their primary imaging modality, with dataset sizes ranging from 10 to 800 scans. The time frames of data collection varied, with some studies having no mention of it, and others reporting periods as recent as 2024; highlighting the up-to-date nature of the review.

The majority of studies focused on the identification and segmentation of missing teeth regions and available bone using CBCT imaging; however, the cross-sectional study by Satapathy et al. [36] evaluated the difference between AI-generated treatment plans and traditionally generated plans. The randomized control trial by Rajan et al. [35] also compared the outcomes of AI-assisted planning with human-generated planning.

Notably, there appears to be more concentration of research on AI’s capabilities in detecting missing teeth and anatomical landmarks, with fewer studies specifically investigating the overall efficiency and clinical impact of AI-driven implant planning processes. This review offers an up-to-date summary of recent findings and points out both advancements and gaps in the current literature.

A range of several different parameters were used to measure the outcomes of the results, and these varied between studies. They commonly included accuracy, precision, recall, F1 score and segmentation overlap metrics such as Dice Similarity Coefficient (DSC) and Jaccard index. These metrics provide a comprehensive insight into the effectiveness, reliability and applicability of the AI models used. See Table 3.

Table 4 below presents a summary of the key outcomes from the studies included in this systematic review, focusing on the performance and clinical effectiveness of various AI technologies used in dental implant planning with CBCT imaging. The table outlines the type of AI software used (DL, CNN), the exact software used (if mentioned) and if additional models were applied (U-net). Whether the systems were overseen or validated by human specialists is also included. Each study’s outcomes are reported in terms of accuracy, precision, recall, success rates, segmentation performance, or comparative clinical metrics. Together, these data illustrate the growing role and effectiveness of AI in enhancing diagnostic accuracy, improving treatment planning efficiency, and supporting clinical decision-making in implant dentistry.

**Table 4:** Outcomes of included studies.

Study	AI Software	Type of AI	Overlooking Method of AI	Outcome of Study
Bayrakdar et al., 2021 [30]	Diagnocat, Inc. (Miami, FL, USA)	3D U-Net DCNN	1 max-fax radiologist with 8 years of experience examined scans and converted them to DICOM	Accuracy of detection: Canal = 72.2%, Sinus and fossa = 66.4%, Missing tooth region = 95.3%. Bone height measurements: no significant difference (*p* > 0.05) Bone thickness measurement showed significant difference (*p* < 0.001)
Gerhardt et al., 2022 [31]	Virtual Patient Creator (Relu BV, Leuven, Belgium—https://creator.relu.eu (accessed on 25 December 2025). 2021)	3D U-Net architecture CNNs	1 dental specialist	Accuracy of detection: Teeth = 99.7%, Missing teeth regions = 99%, Precision of detection: Teeth = 100%. Missing teeth regions = 98.7% Time required: Human = 98 s AI = 1.5 s (*p* < 0.0001)
Al-Sarem et al., 2022 [32]	(1) AlexNet (2) VGG16 (3) VGG19, (4) ResNet50, (5) DenseNet169 (6) MobileNetV3. Additional U-Net model applied to the above systems for tooth segmentation.	3D U-Net CNN	1 GDP with 10 years’ experience to annotate and segment CBCT and 1 Implantologist	Tooth detection: Precision/Recall/F1 score: DenseNet169 = 98/88/93 MobileNetV3 = 95/68/80 VGG19 = 94/75/82 ResNet50 = 94/85/89 VGG16 = 93/87/90 AlexNet = 92/38/54 Missing tooth detection: Precision/Recall/F1 score: DenseNet169 = 89/98/94 MobileNetV3 = 75/97/85 VGG19 = 79/95/86 ResNet50 = 86/95/90 VGG16 = 88/93/90 AlexNet = 61/97/75 U-Net model detection of missing tooth region: Precision = 99% Recall = 97% F1 = 98% U-Net model detection of teeth: Precision = 93% Recall = 98% F1 = 96% Without the application of U-Net segmentation—all the models’ performances were lower.
Bodhe et al., 2022 [33]	N/A	U-net network (CNNs)	1 dentist with over 5 years’ experience.	Performance of U- net results are not coherent—please see note. Missing teeth detection: 751/800 correct Accuracy: Tooth detection: Mandibular = 94.75% Maxillary = 92.71% Alveolar nerve detection = 87.23% Nasal floor detection = 74.51% Maxillary sinus detection = 77.62%
Moufti et al., 2023 [34]	Medical Open Network for Artificial Intelligence (MONAI) framework	U-Net CNN	2 operators and verified by a 3rd specialist in the software	DSC average (measuring overlap of manual/automatic segmentation): Training: 89% Testing: 78% Unilateral edentulous areas = 91% Bilateral cases = 73%
Rajan et al., 2024 [35]	N/A	Deep CNN	‘Experienced clinicians’—no specified number	Success rate: AI = 92% Non AI = 78% (*p* < 0.001) Complications: AI = 8% Non-AI = 18.7% AI interventions required = 0.25 (*p* = 0.015) Average post-operative interventions required: AI: 0.25 Non-AI = 0.47 (*p* = 0.032) Accuracy predictions: AI = 87%
Satapathy et al., 2024 [36]	N/A	Deep learning	1 experienced dentist	Mean deviation: Implant position in relation to structures = 0.5 mm Implant angulation = 0.1 mm Implant depth = 0.1 mm.
Al-Asali et al., 2024 [37]	N/A	Two 2d U- net-based deep learning (CNN) Results presented without/with specialist annotations.	1 oral maxillofacial implantologist with 12 years experience	DSC 0.81/0.93 Jaccard 0.68/0.88 Precision 0.87/0.94 Recall = 0.75/0.93 FPR = 0.00 FNR = 0.25/0.07 VS = 0.15/0.01 HD = 2.96/2.41 MSD = 0.38/0.18 StdSD = 0.41/0.27 HD95 = 1.23/0.66 Volume error rate = 14.32%/1%
Elgarba et al., 2024 [38]	Cloud-based platform (Relu^®^ Creator, Belgium)	3D U- networks	Implant planning: 1 prosthetic dentistry expert, 1 radiology specialist. If needed, then 1 implantologist expert with 20+ years of experience. Quality assessment of implant placement: 12 calibrated dentists through blinded observations with 5 years’ experience	Planning performance assessment: No major corrections: AI = 95% HI = 96% No modifications: AI = 35% HI = 56% Minor adjustments: AI = 60% HI = 40% Turing test observations: 48% of evaluations rated AI as equal to/better than HI. Time efficiency: AI = 198 ± 33 seconds HI = 435 ± 92 seconds. (*p* < 0.05). Median surface deviation (MSD): AI = 0 HI = 0.3 ± 0.17 mm
Alotaibi et al., 2025 [39]	YOLOv11	CNN	N/A	Implant lengths: Accuracy: 76% Precision: 64% Sensitivity: 59% Implant diameter: Accuracy: 59% Precision: 79% Sensitivity: 77%

(DSC = Dice similarity coefficient, FPR = false positive rate, FNR = false negative rate, VS = volumetric similarity, HD = Haudsorff distance, MSD = mean surface distance, StdSD = standard deviation of surface distance, HD95 = 95th percentile of Haudsorff distance).

The studies summarized in Table 5 highlight the strong effectiveness of AI—particularly deep learning (DL) techniques—in dental imaging and treatment planning. With the exception of one study, Rajan et al. [35], all investigations employed convolutional neural networks (CNNs) as a central component of their methodological frameworks.

Out of the ten studies reviewed, eight achieved a CASP score of 70% or higher, indicating strong methodological quality. Six studies [31,32,34,37,38,39] scored 90%, demonstrating rigorous research practices. Conversely, the single RCT by Rajan et al. (2024) [35] scored lower due to vague reporting, and two other studies, Satapathy et al. (2024); Bodhe et al. (2022) [33,36], also lacked clarity, particularly in software disclosure and sample details.

## 4. Discussion

This systematic review aimed to evaluate the role of artificial intelligence (AI), particularly deep learning models applied to CBCT imaging, in dental implant treatment planning. The main findings indicate that AI systems demonstrate high diagnostic accuracy in detecting anatomical structures and edentulous regions, while also significantly improving planning efficiency. The studies included reported performance comparable to, or in some cases exceeding, that of clinicians, particularly in segmentation tasks and treatment planning workflows.

High levels of diagnostic accuracy reported across studies highlight the strength of AI in anatomical detection, particularly for tooth and edentulous region identification. The consistently high performance observed suggests that deep learning models are well-suited to CBCT image analysis, where pattern recognition and segmentation are critical. In addition to accuracy, substantial improvements in time efficiency indicate that AI has the potential to streamline clinical workflows and reduce clinician workload. Gerhardt et al. [31] reported 99.7% accuracy in tooth detection and significant time efficiency (1.5 s vs. 98 s for human planning). Similarly, Bayrakdar et al. [30] found high detection rates for the mandibular canal (72.2%) and missing teeth (95.3%).

Several studies highlighted how expert input and specific model architectures improved outcomes. Al-Sarem et al. [32] showed that adding U-Net to various CNNs significantly boosted performance, with DenseNet169 achieving 94% F1-scores. Al-Asali et al. [37] found expert annotations improved the Dice Similarity Coefficient (DSC) from 0.81 to 0.93. Elgarba et al. [38] demonstrated statistically faster AI-generated plans (198 vs. 435 s), with 95% requiring no major corrections, closely matching human performance (96%).

However, these findings should be interpreted cautiously. The variability in reported accuracy across anatomical structures, such as lower performance in sinus or canal detection, suggests that AI reliability may still depend on anatomical complexity and image quality. Furthermore, the reliance on expert annotation in several studies indicates that optimal AI performance often requires human input, reinforcing the role of AI as a supportive rather than autonomous tool.

There were concerns around inconsistent reporting, limited expert involvement, and ethical oversight. Only four studies explicitly referenced adherence to the Declaration of Helsinki, and two lacked any mention of ethical approval. Some studies also showed data discrepancies or lacked transparency in AI tool specifications, reducing reproducibility. Despite these issues, the overall findings indicate that AI can match or exceed human performance in dental implant planning, though further methodological consistency and ethical compliance are needed for clinical integration. This systematic review synthesised evidence from ten studies exploring the integration of AI particularly convolutional neural networks (CNNs) and deep learning (DL) models into CBCT-based dental implant planning. The collective findings strongly support AI’s emerging role as a powerful tool in diagnostic imaging and treatment planning, with most studies demonstrating high accuracy, efficiency, and clinical relevance of AI-driven segmentation and analysis.

A key finding across the reviewed literature is the efficacy of CNNs and DL models in accurately identifying complex dental anatomical structures such as edentulous areas, adjacent teeth, mandibular canals, and maxillary sinuses Nogueira-Reis et al. Several studies such as Gerhardt et al. and Elgarba et al. [31,38] showed that these systems not only maintained clinical precision but also significantly improved time efficiency, reducing planning time from minutes to mere seconds. This aligns with the broader literature in medicine, where AI-supported diagnostics have also shown enhanced accuracy and reduced workload [40,41,42].

The study by Al-Asali et al. [37] underscored the importance of expert-guided annotations in improving AI model performance, a trend mirrored in other domains of healthcare, where hybrid models, combining AI with clinician input, outperform either in isolation. This is consistent with findings by Raciti et al. [43] and Gulshan et al. [44] in oncology and ophthalmology respectively, where AI has shown diagnostic capabilities equal to or greater than those of clinicians, particularly in image interpretation tasks.

Several reviewed studies highlighted variability in AI performance between the maxilla and mandible. In general, higher accuracy was achieved in mandibular segmentation (DSC: 0.94) compared to maxillary structures (DSC: 0.907) [45]. This likely reflects anatomical complexity: the mandible is denser, better defined, and less variable than the maxilla, which has more intricate features like the nasal floor and sinus cavities [30,33]. The mandibular canal, despite being within the more consistently segmented mandible, remains a challenge for AI due to its small diameter and close proximity to surrounding structures [45]. Nonetheless, AI’s ability to identify such critical landmarks is paramount in minimising surgical risks such as nerve injury, sinus perforation, or root damage [31,32]. Beyond anatomical segmentation, AI has also shown potential in prosthetic design and biomechanical planning. Although not included in this review due to eligibility criteria, studies such as Cho et al. [24] demonstrate how DL models can replicate tooth morphology and design implant-supported crowns with near-human precision. Similarly, Alotaibi et al. [39] showed promising, though still developing, accuracy in AI-predicted implant length and diameter, indicating the potential of AI in supporting but not yet replacing prosthodontic expertise.

Implant placement accuracy is not purely dependent on anatomy; biomechanical factors like angulation, bone quality, occlusion, and aesthetics also play a crucial role in long-term success. Poor planning can lead to peri-implantitis, prosthetic complications, and compromised function [46,47]. AI tools, when integrated into surgical-restorative workflows, offer a means to optimise all these factors systematically.

The Relu^®^ Creator, used in two of the reviewed studies [31,38], exemplifies the practical implementation of AI in dentistry. With FDA approval and cloud-based accessibility, it demonstrates the real-world feasibility of integrating AI into dental workflows. However, like many AI systems, its performance is still highly dependent on input data quality, and human validation remains essential, with only 35% of AI-generated plans requiring no modifications [38].

Technologically, the review also highlights the distinction between 2D U-net and 3D U-net architectures. While 2D U-nets are faster and require less data, they lack the volumetric context necessary for detailed CBCT analysis, making 3D U-nets the preferred choice for high-accuracy segmentation in implant planning [48,49]. Meanwhile, CNNs like VGG, YOLO, and ResNet remain superior for classification and detection, particularly in 2D images like OPGs or bitewings.

Compared to fields like radiology or oncology, AI in dentistry is still underdeveloped, a trend attributed largely to limited, non-standardised datasets and restricted data access due to the decentralised nature of dental practice. In contrast, medical datasets are larger, standardised, and often integrated across national health systems [50,51]. Moreover, current AI systems are not autonomous and should be viewed as decision-support tools, not replacements for clinical judgment. As shown in the reviewed studies, hybrid human-AI collaboration consistently yielded better outcomes than AI or humans alone [37,42].

The responsible use of AI in dentistry also raises ethical and regulatory considerations. Transparency is key: patients should be informed when AI tools are involved in their care, and consent must be obtained [52]. There is also a critical need to avoid bias by ensuring AI models are trained on diverse, representative datasets [53]. Furthermore, the successful integration of AI into dental practice depends heavily on clinician education. Dental professionals must not only learn how to operate AI tools but also understand their limitations, interpret outputs, and know when to override suggestions based on clinical context [54,55]. Without this, the risk of over-reliance or misapplication increases.

## 5. Limitations

In this study there was a relatively small number of included studies, many of which were based on single-centre and limited CBCT datasets, which restricts the generalisability of results and highlights the need for larger, multi-centre investigations. In addition, substantial heterogeneity across studies in terms of AI models, dataset characteristics, annotation methods, and outcome measures limits direct comparability and weakens the overall strength of evidence. Methodological transparency was also inconsistent, with incomplete reporting of data sources, validation processes, and model development in several studies, raising concerns regarding reproducibility and potential bias. Furthermore, variability inherent to CBCT imaging, including differences in resolution, artefacts, and annotation standards, may have influenced AI performance and contributed to inconsistencies in reported outcomes. The absence of standardised performance measures ranging from accuracy and sensitivity to Dice Similarity Coefficient (DSC) and F1-score limits comparability and reduces the strength of collective conclusions. Importantly, most studies focused on technical accuracy rather than clinically meaningful endpoints, and there remains a lack of robust, long-term, real-world validation and regulatory integration. As such, while AI demonstrates promising potential in implant planning, its translation into routine clinical practice remains limited, emphasising the need for standardised evaluation frameworks, improved reporting quality, and clinically driven research. Future research should prioritise the development of large, standardised, and well-annotated CBCT datasets, alongside multi-centre clinical validation studies using unified evaluation metrics.

## 6. Conclusions

AI shows strong potential to enhance CBCT-based dental implant planning by improving diagnostic accuracy, anatomical landmark identification, and workflow efficiency, while reducing operator variability. However, current evidence is limited by small, non-diverse datasets, methodological heterogeneity, and a lack of robust external and prospective clinical validation, restricting generalisability and routine clinical application.

Future research should focus on large-scale, multicentre studies using standardised evaluation frameworks and rigorous validation protocols. Long-term prospective data on safety, reliability, and real-world performance are essential before widespread clinical adoption can be justified.

## Figures and Tables

**Table 1:** (Population, Intervention, Comparison, Outcome) framework.

Component	Description
Pop ulation (P)	Adults aged 18 years and over who had at least one dental implant placed with CBCT imaging used during treatment planning. This age threshold ensured patients were legally autonomous and that implant placement was not influenced by ongoing jaw growth [16]. All genders and languages were included to ensure a representative and inclusive dataset.
Intervention (I)	Use of AI technology during the implant treatment planning stage.
Comparison (C)	Studies comparing AI-assisted planning with conventional CBCT-guided implant planning.
Outcomes (O)	Clinical outcomes related to placed implants, including implant success rate, placement precision, accuracy, and overall treatment effectiveness.

**Table 3:** Metrics table.

Metric	Measures	Formula
Accuracy	Proportion of correct predictions	(TP + TN)/(TP + TN+ FP + FN)
Precision	Proportion of predicted positives that are correct	TP/(TP + FP)
Recall (Sensitivity)	Proportion of actual positives that are correctly identified	TP/(TP + FN)
F1 Score	Harmonic mean of precision and recall	2 × (Precision × recall)/(Precision + recall)
Jaccard index	Ratio of intersection of predicted actual positives to the union of predicted and actual positives	TP/(TP + FP + FN)
Dice similarity Coefficient (DSC)	Degree of overlap between predicted and actual positives	(2 × TP)/(2 × TP + FP + FN)

(TP = true positive, TN = true negative, FP = false positive, FN = false negative).

**Table 5:** Quality assessment table.

Study	Bayra kd ar et al., 2021 [30]	Gerhar dt et al., 2022 [31]	Al-Sarem et al., 2022 [32]	Bodhe et al., 2022 [33]	Moufti et al., 2023 [34]	Rajan et al., 2024 [35]	Satapat hy et al., 2024 [36]	Al-Asali et al., 2024 [37]	Elgar ba et al., 2024 [38]	Alotai bi et al., 2025 [29]
CASP checklist used	CS	CS	CS	CS	CS	RCT	CS	CS	CS	CS
Yes	8	9	9	7	9	8	6	9	9	9
No	0	0	0	0	0	0	0	0	0	0
Can’t tell	2	1	1	3	1	5	4	1	1	1
Not applicable	1	1	1	1	1	0	1	1	1	1
% of Yes	80%	90%	90%	70%	90%	62%	60%	90%	90%	90%

(CS = Cross-sectional study, RCT = Randomised control trial).

## Data Availability

No new data were created or analyzed in this study.

## Supplementary Materials

The following supporting information can be downloaded at: https://www.mdpi.com/article/10.3390/dj14050248/s1, Table S1: PRISMA 2020 Checklist; Table S2: CASP Checklist.
