# Supplementary material for: The Use of Artificial Intelligence in Planning Dental Implant Procedures: A Systematic Review
Source: Dent J (Basel). 2026 Apr 23;14(5):248. doi: 10.3390/dj14050248 (PMC13205848; doi:10.3390/dj14050248)
Supplement: Supplementary file 1 [file dentistry-14-00248-s001.zip › CASP Checklist.pdf]

dentistry journal

MDPI

Appendices

10 CASP Checklists:

1)

CASP

Critical Appraisal Skills Programme

CASP Checklist:

For Descriptive/Cross-Sectional Studies

**Reviewer Name:**	Gulvash Zaman
**Paper Title:**	A deep learning approach for dental implant planning in cone-beam computed tomography images
**Author:**	Bayrakdar et al., 2021
**Web Link:**	https://link.springer.com/article/10.1186/s12880-021-00618-z
**Appraisal Date:**	20/5/2025

Section A: Are the results valid?	
1. Did the study address a clearly focused issue?	Yes. The study evaluated the reliability of an AI system to locate missing teeth regions and structures using CBCT scans.
CONSIDER:<br>*A question can be 'focused' in terms of*<br>• *the population studied*<br>• *the risk factors studied*	

• *is it clear whether the study tried to detect a beneficial or harmful effect*<br>• *the outcomes considered*	
2. Did the authors use an appropriate method to answer their question?	Yes. This study design was appropriate
CONSIDER:<br>• *Is a descriptive/cross-sectional study an appropriate way of answering the question*<br>• *did it address the study question*	
3. Were the subjects recruited in an acceptable way?	Yes, and ethical approval was sought.
CONSIDER:<br>*We are looking for selection bias which might compromise the generalisability of the findings:*<br>• *Was the sample representative of a defined population*<br>• *Was everybody included who should have been included*	
4. Were the measures accurately measured to reduce bias?	Yes. A radiologist and maxillofacial specialist were used to reduce bias.<br>Radiologist also used standardised tools
CONSIDER:<br>*Look for measurement or classification bias:*<br>• *did they use subjective or objective measurements*<br>• *do the measurements truly reflect what you want them to (have they been validated)*	
5. Were the data collected in a way that addressed the research issue?	Yes. The methods were clear and were taken from the same dataset.
CONSIDER:<br>• *if the setting for data collection was justified*<br>• *if it is clear how data were collected (e.g., interview, questionnaire, chart review)*<br>• *if the researcher has justified the methods chosen*<br>• *if the researcher has made the methods explicit (e.g. for interview method, is there an indication of how interviews were conducted?)*	

<table>
<tr><td>6. Did the study have enough participants to minimise the play of chance?</td><td>Can't tell. The sample size is ample, however there is no power calculation mentioned.</td></tr>
<tr><td></td><td></td></tr>
<tr><td colspan="2">CONSIDER:
• if the result is precise enough to make a decision
• if there is a power calculation. This will estimate how many subjects are needed to produce a reliable estimate of the measure(s) of interest.</td></tr>
<tr><td>7. How are the results presented and what is the main result?</td><td>With tables and figures.
The main finding in that Bone height measurements showed no significant difference</td></tr>
<tr><td colspan="2">CONSIDER:
• if, for example, the results are presented as a proportion of people experiencing an outcome, such as risks, or as a measurement, such as mean or median differences, or as survival curves and hazards
• how large this size of result is and how meaningful it is
• how you would sum up the bottom-line result of the trial in one sentence</td></tr>
<tr><td>8. Was the data analysis sufficiently rigorous?</td><td>Yes.
ICC, Bland-Altman metrics used.</td></tr>
<tr><td colspan="2">CONSIDER:
• if there is an in-depth description of the analysis process
• if sufficient data are presented to support the findings</td></tr>
<tr><td>9. Is there a clear statement of findings?</td><td>Yes</td></tr>
<tr><td colspan="2">CONSIDER:
• if the findings are explicit
• if there is adequate discussion of the evidence both for and against the researchers' arguments
• if the researchers have discussed the credibility of their findings
• if the findings are discussed in relation to the original research questions</td></tr>
<tr><td>10. Can the results be applied to the local population?</td><td>Can't tell.
Details of the demographic of the dataset are not at the forefront of the research, but still could provide useful information to draw firm conclusions</td></tr>
</table>

CONSIDER:<br>• *the subjects covered in the study could be sufficiently different from your population to cause concern.*<br>• *your local setting is likely to differ much from that of the study*	
11.How valuable is the research?	Yes. Its valuable as in line with other newer research and coud help to assist in denral implant planning in the future.
CONSIDER:<br>• *one descriptive/cross-sectional study rarely provides sufficiently robust evidence to recommend changes to clinical practice or within health policy decision making*<br>• *if the researcher discusses the contribution the study makes to existing knowledge (e.g., do they consider the findings in relation to current practice or policy, or relevant research-based literature?)*<br>• *if the researchers have discussed whether or how the findings can be transferred to other populations*	

**APPRAISAL SUMMARY**: *List key points from your critical appraisal that need to be considered when assessing the validity of the results and their usefulness in decision-making.*		
**Positive/Methodologically sound**	**Negative/Relatively poor methodology**	**Unknowns**
Clear objectives Appropriate methodology Strong inter-rater reliability Good statistical analysis	No sample size justification	

2)

https://doi.org/10.3390/xxxxx

CASP
Critical Appraisal
Skills Programme

CASP Checklist:

For Descriptive/Cross-Sectional Studies

Reviewer Name:	Gulvash Zaman
Paper Title:	Automated detection and labelling of teeth and small edentulous regions on cone-beam computed tomography using convolutional neural net-works
Author :	Gerhardt et al., 2022
Web Link:	https://www.sciencedirect.com/science/article/pii/S0300571222001956#bib0026
Appraisal Date:	25/4/2025

Section A: Are the results valid?	
12. Did the study address a clearly focused issue?	Yes. The study tested the effectiveness of CNNs to detect and label teeth and accurately segment CBCT scans.
CONSIDER:	

https://doi.org/10.3390/xxxxx

A question can be 'focused' in terms of<br>• *the population studied*<br>• *the risk factors studied*<br>• *is it clear whether the study tried to detect a beneficial or harmful effect*<br>• *the outcomes considered*	
13. Did the authors use an appropriate method to answer their question?	Yes
CONSIDER:<br>• *Is a descriptive/cross-sectional study an appropriate way of answering the question*<br>• *did it address the study question*	
14. Were the subjects recruited in an acceptable way?	Yes. Dataset obtained from Centre of Dentomaxillofacial Radiology of the University Hospitals between March 2016 - January 2021and was ethically approved.
CONSIDER:<br>*We are looking for selection bias which might compromise the generalisability of the findings:*<br>• *Was the sample representative of a defined population*<br>• *Was everybody included who should have been included*	
15. Were the measures accurately measured to reduce bias?	Yes. The outcome was measured using evaluation metrics such as accuracy, Hausdorff distance and Intersection over Union
CONSIDER:<br>*Look for measurement or classification bias:*<br>• *did they use subjective or objective measurements*<br>• *do the measurements truly reflect what you want them to (have they been validated)*	
16. Were the data collected in a way that addressed the research issue?	Yes. Ethical approval was sought.
CONSIDER:<br>• *if the setting for data collection was justified*<br>• *if it is clear how data were collected (e.g., interview, questionnaire, chart review)*<br>• *if the researcher has justified the methods chosen*<br>• *if the researcher has made the methods explicit (e.g. for interview method, is there an indication of how interviews were conducted?)*	

17. Did the study have enough participants to minimise the play of chance?	**Yes. 46 CBCTs used after clinical validation**
CONSIDER:<br>• *if the result is precise enough to make a decision*<br>• *if there is a power calculation. This will estimate how many subjects are needed to produce a reliable estimate of the measure(s) of interest.*	
18. How are the results presented and what is the main result?	That the CNN-based tool used could accurately and precisely detect teeth and edentulous regions.
CONSIDER:<br>• *if, for example, the results are presented as a proportion of people experiencing an outcome, such as risks, or as a measurement, such as mean or median differences, or as survival curves and hazards*<br>• *how large this size of result is and how meaningful it is*<br>• *how you would sum up the bottom-line result of the trial in one sentence*	
19. Was the data analysis sufficiently rigorous?	Yes. Accuracy precision and time were measured. 1 dental specialist overlooked it.
CONSIDER:<br>• *if there is an in-depth description of the analysis process*<br>• *if sufficient data are presented to support the findings*	
20. Is there a clear statement of findings?	Yes.
CONSIDER:<br>• *if the findings are explicit*<br>• *if there is adequate discussion of the evidence both for and against the researchers' arguments*<br>• *if the researchers have discussed the credibility of their findings*<br>• *if the findings are discussed in relation to the original research questions*	
21. Can the results be applied to the local population?	Can't tell.<br>Details of the demographic of the dataset are not at the forefront of the research, but still could provide useful information to draw firm conclusions

CONSIDER:<br>• *the subjects covered in the study could be sufficiently different from your population to cause concern.*<br>• *your local setting is likely to differ much from that of the study*	
22.How valuable is the research?	Very valuable. Gives us accuracy of detection, and precision of detection of teeth and missing teeth, as well as time efficiency with statistical analysis.

CONSIDER:

- *one descriptive/cross-sectional study rarely provides sufficiently robust evidence to recommend changes to clinical practice or within health policy decision making*
- *if the researcher discusses the contribution the study makes to existing knowledge (e.g., do they consider the findings in relation to current practice or policy, or relevant research-based literature?)*
- *if the researchers have discussed whether or how the findings can be transferred to other popula-*

**APPRAISAL SUMMARY**: *List key points from your critical appraisal that need to be considered when assessing the validity of the results and their usefulness in decision-making.*		
**Positive/Methodologically sound**	**Negative/Relatively poor methodology**	**Unknowns**
**Good methodology.**<br><br>**Clear results with detailed analysis**	**Lack of explicit potential cofound-ers.**	

3)

CASP
Critical Appraisal
Skills Programme

CASP Checklist:

For Descriptive/Cross-Sectional Studies

Reviewer Name:	Gulvash Zaman
Paper Title:	Enhanced Tooth Region Detection Using Pretrained Deep Learning Models.
Author:	Al-Sarem et al., 2022
Web Link:	https://pmc.ncbi.nlm.nih.gov/articles/PMC9692549/
Appraisal Date:	25/4/25

Section A: Are the results valid?	
23. Did the study address a clearly focused issue?	Yes. It aimed to evaluate the effectiveness of 6 AI models in detecting missing teeth.
CONSIDER:<br>*A question can be 'focused' in terms of*<br>• *the population studied*<br>• *the risk factors studied*<br>• *is it clear whether the study tried to detect a beneficial or harmful effect*<br>• *the outcomes considered*	
24. Did the authors use an appropriate method to answer their question?	Yes.
CONSIDER:<br>• *Is a descriptive/cross-sectional study an appropriate way of answering the question*<br>• *did it address the study question*	
25. Were the subjects recruited in an acceptable way?	Yes. They were processed and divided into 3 groups: training, validation and testing.
CONSIDER:<br>*We are looking for selection bias which might compromise the generalisability of the findings:*<br>• *Was the sample representative of a defined population*<br>• *Was everybody included who should have been included*	

26. Were the measures accurately measured to reduce bias?	Yes. Accuracy, precision, recall, F1 score and MCC were used.
CONSIDER:<br>*Look for measurement or classification bias:*<br>• *did they use subjective or objective measurements*<br>• *do the measurements truly reflect what you want them to (have they been validated)*	
27. Were the data collected in a way that addressed the research issue?	Yes. The collection of data was standardised.
CONSIDER:<br>• *if the setting for data collection was justified*<br>• *if it is clear how data were collected (e.g., interview, questionnaire, chart review)*<br>• *if the researcher has justified the methods chosen*<br>• *if the researcher has made the methods explicit (e.g. for interview method, is there an indication of how interviews were conducted?)*	
28. Did the study have enough participants to minimise the play of chance?	Yes. 500 CBCTs
CONSIDER:<br>• *if the result is precise enough to make a decision*<br>• *if there is a power calculation. This will estimate how many subjects are needed to produce a reliable estimate of the measure(s) of interest.*	
29. How are the results presented and what is the main result?	Presented in tables and with detailed performance metrics.
CONSIDER:<br>• *if, for example, the results are presented as a proportion of people experiencing an outcome, such as risks, or as a measurement, such as mean or median differences, or as survival curves and hazards*<br>• *how large this size of result is and how meaningful it is*<br>• *how you would sum up the bottom-line result of the trial in one sentence*	

30.Was the data analysis sufficiently rigorous?	Yes. Statistical methods were used to provide information on each AI model.
CONSIDER:<br>• *if there is an in-depth description of the analysis process*<br>• *if sufficient data are presented to support the findings*	
31.Is there a clear statement of findings?	Yes. Clear and comprehensive discussion on the findings.
CONSIDER:<br>• *if the findings are explicit*<br>• *if there is adequate discussion of the evidence both for and against the researchers' arguments*<br>• *if the researchers have discussed the credibility of their findings*<br>• *if the findings are discussed in relation to the original research questions*	
32.Can the results be applied to the local population?	Can't tell.<br>Details of the demographic of the dataset are not at the forefront of the research, but still could provide useful information to draw firm conclusions
CONSIDER:<br>• *the subjects covered in the study could be sufficiently different from your population to cause concern.*<br>• *your local setting is likely to differ much from that of the study*	
33.How valuable is the research?	Yes. Valuable as it gives an insight into the CNNs being trialled in implant planning.
CONSIDER:<br>• *one descriptive/cross-sectional study rarely provides sufficiently robust evidence to recommend changes to clinical practice or within health policy decision making*<br>• *if the researcher discusses the contribution the study makes to existing knowledge (e.g., do they consider the findings in relation to current practice or policy, or relevant research-based literature?)*<br>• *if the researchers have discussed whether or how the findings can be transferred to other populations*	
**APPRAISAL SUMMARY**: ***List key points from your critical appraisal that need to be considered when assessing the validity of the results and their usefulness in decision-making.***	

Positive/Methodologically sound	Negative/Relatively poor methodology	Unknowns
Clear research question Appropriate design and methodology<br>Use of objective performance metrics<br>Detailed results		

4)

CASP
Critical Appraisal
Skills Programme

CASP Checklist:

For Descriptive/Cross-Sectional Studies

Reviewer Name:	Gulvash Zaman
Paper Title:	Design and development of deep learning approach for dental implant planning.
Author:	Bodhe et al., 2022
Web Link:	https://www.semanticscholar.org/paper/Design-and- Development-of-Deep-Learning-Approach-Bodhe- Sivakumar/870de7ef71520a041ce007c1f1a50e6efc07279f
Appraisal Date:	20/4/25

Section A: Are the results valid?	
34. Did the study address a clearly focused issue?	Yes. The focus of the study was to evaluate a deep learning model for implant planning, using CBCTs.
CONSIDER:<br>*A question can be 'focused' in terms of*<br>• *the population studied*<br>• *the risk factors studied*<br>• *is it clear whether the study tried to detect a beneficial or harmful effect*	
• *the outcomes considered*	
35. Did the authors use an appropriate method to answer their question?	Yes. The study design was appropriate.
CONSIDER:<br>• *Is a descriptive/cross-sectional study an appropriate way of answering the question*<br>• *did it address the study question*	
36. Were the subjects recruited in an acceptable way?	Can't tell.<br>No mention of ethics approval. No clear information on how the data set was selected.
CONSIDER:<br>*We are looking for selection bias which might compromise the generalisability of the findings:*<br>• *Was the sample representative of a defined population*<br>• *Was everybody included who should have been included*	
37. Were the measures accurately measured to reduce bias?	Yes. Accuracy, precision, sensitivity, specificity were all used.
CONSIDER:<br>*Look for measurement or classification bias:*<br>• *did they use subjective or objective measurements*<br>• *do the measurements truly reflect what you want them to (have they been validated)*	

38. Were the data collected in a way that addressed the research issue?	Yes. Data set collected from the same clinic.
CONSIDER:<br>• *if the setting for data collection was justified*<br>• *if it is clear how data were collected (e.g., interview, questionnaire, chart review)*<br>• *if the researcher has justified the methods chosen*<br>• *if the researcher has made the methods explicit (e.g. for interview method, is there an indication of how interviews were conducted?)*	
39. Did the study have enough participants to minimise the play of chance?	Yes, 500 patients, 800 CBCTs
CONSIDER:<br>• *if the result is precise enough to make a decision*<br>• *if there is a power calculation. This will estimate how many subjects are needed to produce a reliable estimate of the measure(s) of interest.*	
40. How are the results presented and what is the main result?	Results present with clear performance metrics, however there are some errors in the table that don’t match the text.
CONSIDER:<br>• *if, for example, the results are presented as a proportion of people experiencing an outcome, such as risks, or as a measurement, such as mean or median differences, or as survival curves and hazards*<br>• *how large this size of result is and how meaningful it is*<br>• *how you would sum up the bottom-line result of the trial in one sentence*	
41. Was the data analysis sufficiently rigorous?	Yes
CONSIDER:<br>• *if there is an in-depth description of the analysis process*<br>• *if sufficient data are presented to support the findings*	

42.Is there a clear statement of findings?	Yes, the conclusion was that the models could accurately detect regions for dental implants.
CONSIDER:<br>• *if the findings are explicit*<br>• *if there is adequate discussion of the evidence both for and against the researchers' arguments*<br>• *if the researchers have discussed the credibility of their findings*<br>• *if the findings are discussed in relation to the original research questions*	
43.Can the results be applied to the local population?	Can't tell.<br>Lack of timeframe of dataset collection.<br>Details of the demographic of the dataset are not at the forefront of the research, but still could provide useful information to draw firm conclusions.
CONSIDER:<br>• *the subjects covered in the study could be sufficiently different from your population to cause concern.*<br>• *your local setting is likely to differ much from that of the study*	
44.How valuable is the research?	Can't say.<br>Although a detailed analysis of the models performance was given – information about the model is lacking.<br>Some discrepancies between the table and main text

CONSIDER:
- *one descriptive/cross-sectional study rarely provides sufficiently robust evidence to recommend changes to clinical practice or within health policy decision making*
- *if the researcher discusses the contribution the study makes to existing knowledge (e.g., do they consider the findings in relation to current practice or policy, or relevant research-based literature?)*
- *if the researchers have discussed whether or how the findings can be transferred to other popula-*

**APPRAISAL SUMMARY**: *List key points from your critical appraisal that need to be considered when assessing the validity of the results and their usefulness in decision-making.*		
**Positive/Methodologically sound**	**Negative/Relatively poor methodology**	**Unknowns**

**Well analysed.**	**No time frame of data collection Discrepancies in results/data Details on AI software not given**	

5)

CASP
Critical Appraisal
Skills Programme

CASP Checklist:

For Descriptive/Cross-Sectional Studies

Reviewer Name:	Gulvash Zaman
Paper Title:	Developing an Artificial Intelligence Solution to Autosegment the Edentulous Mandibular Bone for Implant Planning.
Author:	Moufti et al., 2023
Web Link:	https://www.thieme- connect.com/products/ejournals/html/10.1055/s-0043-1764425
Appraisal Date:	20/4/25

**Section A: Are the results valid?**	
45. Did the study address a clearly focused issue?	Yes. The study aimed to test AI performance in implant planning, design, missing teeth detection and success prediction.

CONSIDER:<br>*A question can be 'focused' in terms of*<br>• *the population studied*<br>• *the risk factors studied*<br>• *is it clear whether the study tried to detect a beneficial or harmful effect*	
• *the outcomes considered*	
46. Did the authors use an appropriate method to answer their question?	Yes.
CONSIDER:<br>• *Is a descriptive/cross-sectional study an appropriate way of answering the question*<br>• *did it address the study question*	
47. Were the subjects recruited in an acceptable way?	Yes. From University dental hospital, Sharjah with a clear inclusion criteria to maintain homogenous results.
CONSIDER:<br>*We are looking for selection bias which might compromise the generalisability of the findings:*<br>• *Was the sample representative of a defined population*<br>• *Was everybody included who should have been included*	
48. Were the measures accurately measured to reduce bias?	Yes.<br>ITK-snap software used to manually segment and Dice Similarity Coefficient to compare.
CONSIDER:<br>*Look for measurement or classification bias:*<br>• *did they use subjective or objective measurements*<br>• *do the measurements truly reflect what you want them to (have they been validated)*	
49. Were the data collected in a way that addressed the research issue?	Yes. Data collection was standardised and split into training and testing.
CONSIDER:<br>• *if the setting for data collection was justified*<br>• *if it is clear how data were collected (e.g., interview, questionnaire, chart review)*<br>• *if the researcher has justified the methods chosen*<br>• *if the researcher has made the methods explicit (e.g. for interview method, is there an indication of how interviews were conducted?)*	

50. Did the study have enough participants to minimise the play of chance?	Yes.<br>43 cases were used.
CONSIDER:<br>• *if the result is precise enough to make a decision*<br>• *if there is a power calculation. This will estimate how many subjects are needed to produce a reliable estimate of the measure(s) of interest.*	
51. How are the results presented and what is the main result?	**Results presented using the DSC in clear tables. 83%.**
CONSIDER:<br>• *if, for example, the results are presented as a proportion of people experiencing an outcome, such as risks, or as a measurement, such as mean or median differences, or as survival curves and hazards*<br>• *how large this size of result is and how meaningful it is*<br>• *how you would sum up the bottom-line result of the trial in one sentence*	
52. Was the data analysis sufficiently rigorous?	**Yes. Two operators and a 3rd specialist in the software validated results.**
CONSIDER:<br>• *if there is an in-depth description of the analysis process*<br>• *if sufficient data are presented to support the findings*	
53. Is there a clear statement of findings?	Yes.
CONSIDER:<br>• *if the findings are explicit*<br>• *if there is adequate discussion of the evidence both for and against the researchers' arguments*<br>• *if the researchers have discussed the credibility of their findings*<br>• *if the findings are discussed in relation to the original research questions*	
54. Can the results be applied to the local population?	Can't tell.<br>Details of the demographic of the dataset are not at the forefront of the research, but still could provide useful information to draw firm conclusions

CONSIDER:<br>• *the subjects covered in the study could be sufficiently different from your population to cause concern.*<br>• *your local setting is likely to differ much from that of the study*	
55.How valuable is the research?	Yes.
CONSIDER:<br>• *one descriptive/cross-sectional study rarely provides sufficiently robust evidence to recommend changes to clinical practice or within health policy decision making*	

- *if the researcher discusses the contribution the study makes to existing knowledge (e.g., do they consider the findings in relation to current practice or policy, or relevant research-based literature?)*
- *if the researchers have discussed whether or how the findings can be transferred to other popula-* [illegible]

**APPRAISAL SUMMARY**: *List key points from your critical appraisal that need to be considered when assessing the validity of the results and their usefulness in decision-making.*		
**Positive/Methodologically sound**	**Negative/Relatively poor methodology**	**Unknowns**
**Clearly defined research question**<br>**Appropriate validation methods for collection and analysis**<br>**Use of DSC**<br>**Transparent reporting of findings**<br>**Ethical approval**	**No mention of time frame of collection**<br><br>**Small sample**	

6)

CASP Checklist:

CASP Critical Appraisal Skills Programme

For Randomised Controlled Trials (RCTs)

**Reviewer Name:**	Gulvash Zaman
**Paper Title:**	Evaluating the Role of AI in Predicting the Success of Dental Implants Based on Preoperative CBCT Images: A Randomized Controlled Trial.
**Author:**	Rajan et al., 2024
**Web Link:**	https://pmc.ncbi.nlm.nih.gov/articles/PMC11000953/
**Appraisal Date:**	20/4/25

Section A Is the basic study design valid for a randomised controlled trial?	
56. Did the study address a clearly formulated research question?	Yes<br>Population = 18-70 years old who needed an implant<br>Intervention = AI predictions Comparison = traditional assessment predictions<br>Outcome = success / complications
CONSIDER:<br>*Was the study designed to assess the outcomes of an intervention?*<br>*Is the research question 'formulated' in terms of:*<br>• *Population studied*<br>• *Intervention given*<br>• *Comparator chosen*<br>• *Outcomes measured?*	

57. Was the assignment of participants to interventions randomised?	Yes – computer generated randomisation
CONSIDER:<br>• *How was randomisation carried out? Was the method appropriate?*<br>• *Was randomisation sufficient to eliminate systematic bias?*<br>• *Was the allocation sequence concealed from investigators and participants?*	
58. Were all participants who entered the study accounted for at its conclusion?	Yes. All 150 were reported on in the results
CONSIDER:<br>• *Were losses to follow-up and exclusions after randomisation accounted for?*<br>• *Were participants analysed in the study groups to which they were randomised (intention-to-treat analysis)?*<br>• *Was the study stopped early? If so, what was the reason?*	
Section B Was the study methodologically sound?	
59. (a) Were the participants 'blind' to intervention they were given?	Can't tell
(b) Were the investigators 'blind' to the intervention they were giving to participants?	Can't tell
(c) Were the people assessing/analysing outcome/s 'blinded'?	Can't tell
60. Were the study groups similar at the start of the randomised controlled trial?	Can't tell

CONSIDER:<br>• *Were the baseline characteristics of each study group (e.g. age, sex, socio-economic group) clearly set out?*<br>• *Were there any differences between the study groups that could affect the outcome/s?*	
61. Apart from the experimental intervention, did each study group receive the same level of care (that is, were they treated equally)?	Yes. Both groups underwent CBCTs pre-operatively.
CONSIDER:<br>• *Was there a clearly defined study protocol?*<br>• *If any additional interventions were given (e.g. tests or treatments), were they similar between the study groups?*<br>• *Were the follow-up intervals the same for each study group?*	
Section C: What are the results?	
62.Were the effects of intervention reported comprehensively?	Yes
CONSIDER:<br>• *Was a power calculation undertaken?*<br>• *What outcomes were measured, and were they clearly specified?*<br>• *How were the results expressed? For binary outcomes, were relative and absolute effects reported?*<br>• *Were the results reported for each outcome in each study group at each follow-up interval?*<br>• *Was there any missing or incomplete data?*<br>• *Was there differential drop-out between the study groups that could affect the results?*<br>• *Were potential sources of bias identified?*<br>• *Which statistical tests were used?*<br>• *Were p values reported?*	

63. Was the precision of the estimate of the intervention or treatment effect reported?	Yes – partially. The study reports statistical significance but the confidence intervals have not been reported on – limiting the precision.
CONSIDER:	
• *Were confidence intervals (CIs) reported?*	
64. Do the benefits of the experimental intervention outweigh the harms and costs?	Yes – the clinical outcomes are all relevant (success rate, complication rates)
CONSIDER:<br>• *What was the size of the intervention or treatment effect?*<br>• *Were harms or unintended effects reported for each study group?*<br>• *Was a cost-effectiveness analysis undertaken? (Cost-effectiveness analysis allows a comparison to be made between different interventions used in the care of the same condition or problem.)*	
Section D: Will the results help locally?	
65. Can the results be applied to your local population/in your context?	Can't tell.<br>Details of the demographic of the dataset are not at the forefront of the research, but still could provide useful information to draw firm conclusions
CONSIDER:<br>• *Are the study participants similar to the people in your care?*<br>• *Would any differences between your population and the study participants alter the outcomes reported in the study?*<br>• *Are the outcomes important to your population?*<br>• *Are there any outcomes you would have wanted information on that have not been studied or reported?*<br>• *Are there any limitations of the study that would affect your decision?*	

66. Would the experimental intervention provide greater value to the people in your care than any of the existing interventions?	Yes.
CONSIDER:<br>• *What resources are needed to introduce this intervention taking into account time, finances, and skills development or training needs?*<br>• *Are you able to disinvest resources in one or more existing interventions in order to be able to re-invest in the new intervention?*	

APPRAISAL SUMMARY: *List key points from your critical appraisal that need to be considered when assessing the validity of the results and their usefulness in decision-making.*

**Positive/Methodologically sound**	**Negative/Relatively poor methodology**	**Unknowns**
**Good research question. Randomised allocation of groups. All results accounted for.**	**Not enough detail in exactly how data was extracted and measured.**<br><br>**No details on the experience of clinicians assessing data.**<br><br>**No details on AI method used.**	**Medical conditions that could affect results not specified.**<br><br>**Timeframe of CBCT collection not specified.**

7)

CASP

Critical Appraisal
Skills Programme

CASP Checklist:

For Descriptive/Cross-Sectional Studies

Reviewer Name:	Gulvash Zaman
Paper Title:	AI-Assisted Treatment Planning for Dental Implant Placement: Clinical vs AI-Generated Plans
Author:	Satapathy et al., 2024
Web Link:	https://pmc.ncbi.nlm.nih.gov/articles/PMC11000953/
Appraisal Date:	21/4/25

Section A: Are the results valid?	
67. Did the study address a clearly focused issue?	Yes.<br>A deep learning software was used to create a treatment plan for implants and then compared with those created by humans.
CONSIDER:<br>*A question can be 'focused' in terms of*<br>• *the population studied*	
• *the risk factors studied*<br>• *is it clear whether the study tried to detect a beneficial or harmful effect*<br>• *the outcomes considered*	
68. Did the authors use an appropriate method to answer their question?	Yes – a cross sectional design was used using pre-existing CBCT data
CONSIDER:<br>• *Is a descriptive/cross-sectional study an appropriate way of answering the question*<br>• *did it address the study question*	
69. Were the subjects recruited in an acceptable way?	Can't tell.<br>20 CBCTs were collected from patients based off an inclusion criteria. However no mention of the institution it was collected from or the timeframe is mentioned.

CONSIDER:<br>*We are looking for selection bias which might compromise the generalisability of the findings:*<br>• *Was the sample representative of a defined population*<br>• *Was everybody included who should have been included*	
70. Were the measures accurately measured to reduce bias?	Yes. Implant position, angulation and depth were quantitively assessed
CONSIDER:<br>*Look for measurement or classification bias:*<br>• *did they use subjective or objective measurements*<br>• *do the measurements truly reflect what you want them to (have they been validated)*	
71. Were the data collected in a way that addressed the research issue?	Yes – CBCT data was analysed appropriately and comparisons were made.
CONSIDER:<br>• *if the setting for data collection was justified*<br>• *if it is clear how data were collected (e.g., interview, questionnaire, chart review)*<br>• *if the researcher has justified the methods chosen*<br>• *if the researcher has made the methods explicit (e.g. for interview method, is there an indication of how interviews were conducted?)*	
72. Did the study have enough participants to minimise the play of chance?	Cant tell - 20 participants present with no mention of where they were from, and when the sample was collected.
CONSIDER:	
• ***if the result is precise enough to make a decision***<br>• ***if there is a power calculation. This will estimate how many subjects are needed to produce a reliable estimate of the measure(s) of interest.***	
73.How are the results presented and what is the main result?	AI generated plans were similar to clinician generated plans with mean deviation of 0.5mm in the implant position in relation to anatomical structures.
CONSIDER:<br>• *if, for example, the results are presented as a proportion of people experiencing an outcome, such as risks, or as a measurement, such as mean or median differences, or as survival curves and hazards*<br>• *how large this size of result is and how meaningful it is*	

• *how you would sum up the bottom-line result of the trial in one sentence*	
74.Was the data analysis sufficiently rigorous?	Can’t tell – as results weren’t explained in detail.
CONSIDER:<br>• *if there is an in-depth description of the analysis process*<br>• *if sufficient data are presented to support the findings*	
75.Is there a clear statement of findings?	Yes
CONSIDER:<br>• *if the findings are explicit*<br>• *if there is adequate discussion of the evidence both for and against the researchers’ arguments*<br>• *if the researchers have discussed the credibility of their findings*<br>• *if the findings are discussed in relation to the original research questions*	
76.Can the results be applied to the local population?	Can’t tell.<br>Details of the demographic of the dataset are not at the forefront of the research, but still could provide useful information to draw firm conclusions
CONSIDER:<br>• *the subjects covered in the study could be sufficiently different from your population to cause concern.*<br>• *your local setting is likely to differ much from that of the study*	
77.How valuable is the research?	Valuable as it suggests that AI assisted planning can match clinicians.

CONSIDER:

- *one descriptive/cross-sectional study rarely provides sufficiently robust evidence to recommend changes to clinical practice or within health policy decision making*
- *if the researcher discusses the contribution the study makes to existing knowledge (e.g., do they consider the findings in relation to current practice or policy, or relevant research-based literature?)*
- *if the researchers have discussed whether or how the findings can be transferred to other popula-*

**APPRAISAL SUMMARY**: *List key points from your critical appraisal that need to be considered when assessing the validity of the results and their usefulness in decision-making.*		
**Positive/Methodologically sound**	**Negative/Relatively poor methodology**	**Unknowns**
**Clear methods and objectives**<br>**Quantitative assessment Findings are consistent with literature**	**Limited confounding factors**<br>**Not much details on dataset**<br>**No detail on type of AI software used.**<br>**Lack of ethical approval**	

8)

Critical Appraisal Skills Programme

CASP Checklist:

For Descriptive/Cross-Sectional Studies

Reviewer Name:	Gulvash Zaman
Paper Title:	Deep learning-based approach for 3D bone segmentation and prediction of missing tooth region for dental implant planning.
Author:	Al-Asali et al., 2024
Web Link:	https://www.nature.com/articles/s41598-024-64609-0
Appraisal Date:	21/4/25

<table>
<tr><td colspan="2">Section A: Are the results valid?</td></tr>
<tr><td>78. Did the study address a clearly focused issue?</td><td>Yes.It aimed to enhance the efficiency and precision of dental implant placement by segmenting CBCT scans using AI.</td></tr>
<tr><td colspan="2">CONSIDER:
A question can be ‘focused’ in terms of
• the population studied
• the risk factors studied
• is it clear whether the study tried to detect a beneficial or harmful effect
• the outcomes considered</td></tr>
<tr><td>79. Did the authors use an appropriate method to answer their question?</td><td>Yes.</td></tr>
<tr><td></td><td></td></tr>
<tr><td colspan="2">CONSIDER:
• Is a descriptive/cross-sectional study an appropriate way of answering the question
• did it address the study question</td></tr>
<tr><td>80. Were the subjects recruited in an acceptable way?</td><td>Yes. 150 CBCT scans from 2018-2023, aged 16-72 from Taibah Dental Hospital</td></tr>
<tr><td colspan="2">CONSIDER:
We are looking for selection bias which might compromise the generalisability of the findings:
• Was the sample representative of a defined population
• Was everybody included who should have been included</td></tr>
<tr><td>81. Were the measures accurately measured to reduce bias?</td><td>Yes. An experienced implantologist annotated the results. Also Dice coefficient, precision and recall were used</td></tr>
<tr><td colspan="2">CONSIDER:
Look for measurement or classification bias:

• did they use subjective or objective measurements
• do the measurements truly reflect what you want them to (have they been validated)</td></tr>
</table>

82. Were the data collected in a way that addressed the research issue?	Yes.
CONSIDER:<br>• *if the setting for data collection was justified*<br>• *if it is clear how data were collected (e.g., interview, questionnaire, chart review)*<br>• *if the researcher has justified the methods chosen*<br>• *if the researcher has made the methods explicit (e.g. for interview method, is there an indication of how interviews were conducted?)*	
83. Did the study have enough participants to minimise the play of chance?	Yes. 890 initial scans narrowed down to 150
CONSIDER:<br>• *if the result is precise enough to make a decision*<br>• *if there is a power calculation. This will estimate how many subjects are needed to produce a reliable estimate of the measure(s) of interest.*	
84.How are the results presented and what is the main result?	Quantitative metrics are used to present results. The obtained dice, Jaccard, precision, and recall values were 0.93, 0.88, 0.94, and 0.93, respectively.<br>Volume error rate was low (1%)
CONSIDER:<br>• *if, for example, the results are presented as a proportion of people experiencing an outcome, such as risks, or as a measurement, such as mean or median differences, or as survival curves and hazards*<br>• *how large this size of result is and how meaningful it is*<br>• *how you would sum up the bottom-line result of the trial in one sentence*	
85.Was the data analysis sufficiently rigorous?	Yes. Comprehensive evaluation and use of an expert with12 years' experience.
CONSIDER:<br>• *if there is an in-depth description of the analysis process*<br>• *if sufficient data are presented to support the findings*	

<table>
<tr><td>86.Is there a clear statement of findings?</td><td>Yes.</td></tr>
<tr><td colspan="2">CONSIDER:
• if the findings are explicit
• if there is adequate discussion of the evidence both for and against the researchers' arguments
• if the researchers have discussed the credibility of their findings
• if the findings are discussed in relation to the original research questions</td></tr>
<tr><td>87.Can the results be applied to the local population?</td><td>Can't tell.
Details of the demographic of the dataset are not at the forefront of the research, but still could provide useful information to draw firm conclusions</td></tr>
<tr><td colspan="2">CONSIDER:
• the subjects covered in the study could be sufficiently different from your population to cause concern.
• your local setting is likely to differ much from that of the study</td></tr>
<tr><td>88.How valuable is the research?</td><td>Contributes to advancement in AI and to the existing research.</td></tr>
<tr><td colspan="2">CONSIDER:
• one descriptive/cross-sectional study rarely provides sufficiently robust evidence to recommend changes to clinical practice or within health policy decision making</td></tr>
</table>

- *if the researcher discusses the contribution the study makes to existing knowledge (e.g., do they consider the findings in relation to current practice or policy, or relevant research-based literature?)*
- *if the researchers have discussed whether or how the findings can be transferred to other popula-* [illegible]

<table>
<tr><td colspan="3">APPRAISAL SUMMARY: List key points from your critical appraisal that need to be considered when assessing the validity of the results and their usefulness in decision-making.</td></tr>
<tr><td>Positive/Methodologically sound</td><td>Negative/Relatively poor methodology</td><td>Unknowns</td></tr>
</table>

**Clearly defined results and outcomes**<br>**Appropriate use of metrics**<br>**High accuracy**<br>**Detailed methodology**		

9)

CASP

Critical Appraisal
Skills Programme

CASP Checklist:

For Descriptive/Cross-Sectional Studies

Reviewer Name:	Gulvash Zaman
Paper Title:	Novel AI-based automated virtual implant placement: Artificial versus Human Intelligence.
Author:	Elgarba et al., 2024
Web Link:	https://www.sciencedirect.com/science/article/pii/S0300571224003154
Appraisal Date:	21/4/25

Section A: Are the results valid?

89. Did the study address a clearly focused issue?	Yes. The study aimed to assess the acceptance of an AI tool for implant placement in single mandibular edentulous premolar/molar regions by comparing its performance with a human expert.
CONSIDER:<br>*A question can be 'focused' in terms of*<br>• *the population studied*<br>• *the risk factors studied*<br>• *is it clear whether the study tried to detect a beneficial or harmful effect*<br>• *the outcomes considered*	
90. Did the authors use an appropriate method to answer their question?	Yes.
CONSIDER:<br>• *Is a descriptive/cross-sectional study an appropriate way of answering the question*<br>• *did it address the study question*	
91. Were the subjects recruited in an acceptable way?	Yes. 10 CBCT and IO scans from UZ Leuven Hospital
CONSIDER:<br>*We are looking for selection bias which might compromise the generalisability of the findings:*<br>• *Was the sample representative of a defined population*<br>• *Was everybody included who should have been included*	
92. Were the measures accurately measured to reduce bias?	Yes. Evaluations conducted by 12 calibrated dentists through blinded observations using a visual analogue scale.
CONSIDER:<br>*Look for measurement or classification bias:*<br>• *did they use subjective or objective measurements*<br>• *do the measurements truly reflect what you want them to (have they been validated)*	
93. Were the data collected in a way that addressed the research issue?	Yes. The collection of data involved standardised assessments of implants plans, time efficiency measurements.

CONSIDER:<br>• *if the setting for data collection was justified*<br>• *if it is clear how data were collected (e.g., interview, questionnaire, chart review)*<br>• *if the researcher has justified the methods chosen*	
• *if the researcher has made the methods explicit (e.g. for interview method, is there an indication of how interviews were conducted?)*	
94. Did the study have enough participants to minimise the play of chance?	Yes. 360 observations – 10 CBCTs
CONSIDER:<br>• *if the result is precise enough to make a decision*<br>• *if there is a power calculation. This will estimate how many subjects are needed to produce a reliable estimate of the measure(s) of interest.*	
95. How are the results presented and what is the main result?	In tables.<br>AI completed planning more than twice as fast as HI, taking only 198 ± 33 s compared to 435 ± 92 s ($p < 0.05$).
CONSIDER:<br>• *if, for example, the results are presented as a proportion of people experiencing an outcome, such as risks, or as a measurement, such as mean or median differences, or as survival curves and hazards*<br>• *how large this size of result is and how meaningful it is*<br>• *how you would sum up the bottom-line result of the trial in one sentence*	
96. Was the data analysis sufficiently rigorous?	Yes. Statistical analysis was employed, including standard deviations, and significance testing.
CONSIDER:<br>• *if there is an in-depth description of the analysis process*<br>• *if sufficient data are presented to support the findings*	
97. Is there a clear statement of findings?	Yes. AI is more efficient than HI
CONSIDER:<br>• *if the findings are explicit*<br>• *if there is adequate discussion of the evidence both for and against the researchers' arguments*<br>• *if the researchers have discussed the credibility of their findings*	

• *if the findings are discussed in relation to the original research questions*	
98.Can the results be applied to the local population?	Can't tell.<br>Details of the demographic of the dataset are not at the forefront of the research, but still could provide useful information to draw firm conclusions
CONSIDER:<br>• *the subjects covered in the study could be sufficiently different from your population to cause concern.*	
• *your local setting is likely to differ much from that of the study*	
99.How valuable is the research?	Yes. This research provides valuable insights into the potential of AI planning
CONSIDER:<br>• *one descriptive/cross-sectional study rarely provides sufficiently robust evidence to recommend changes to clinical practice or within health policy decision making*<br>• *if the researcher discusses the contribution the study makes to existing knowledge (e.g., do they consider the findings in relation to current practice or policy, or relevant research-based literature?)*<br>• *if the researchers have discussed whether or how the findings can be transferred to other populations*	

**APPRAISAL SUMMARY**: *List key points from your critical appraisal that need to be considered when assessing the validity of the results and their usefulness in decision-making.*		
**Positive/Methodologically sound**	**Negative/Relatively poor methodology**	**Unknowns**
**Clear objective**<br>**Clear methods**<br>**Clear study design and results**<br>**Blinded assessment reducing bias**<br>**Use of statistical analysis**	**Sample size**	

10)

CASP
Critical Appraisal
Skills Programme

CASP Checklist:

For Descriptive/Cross-Sectional Studies

Reviewer Name:	Gulvash Zaman
Paper Title:	Automatic placement of simulated dental implants within CBCT images in optimum positions: a deep learning model
Author:	Alotaibi et al., 2025
Web Link:	https://link.springer.com/article/10.1007/s11517-025-03327-9
Appraisal Date:	21/4/25

During critical appraisal, never make assumptions about what the researchers have done. If it is not possible to tell, use the "Can't tell" response box. If you can't tell, at best it means the researchers have not been explicit or transparent, but at worst it could mean the researchers have not undertaken a particular task or process. Once you've finished the critical appraisal, if there are a large number of "Can't tell" responses, consider whether the findings of the study are trustworthy and interpret the results with caution.

Section A: Are the results valid?

100. Did the study address a clearly focused issue?	Yes. The study aims to evaluate an AI model to plan implant size and position

CONSIDER:<br>*A question can be 'focused' in terms of*<br>• *the population studied*<br>• *the risk factors studied*<br>• *is it clear whether the study tried to detect a beneficial or harmful effect*<br>• *the outcomes considered*	
101. Did the authors use an appropriate method<br>to answer their question?	Yes.
CONSIDER:<br>• *Is a descriptive/cross-sectional study an appropriate way of answering the question*<br>• *did it address the study question*	
102. Were the subjects recruited in an acceptable way?	Yes. Ethical approval sought, and scans selected from King Saud University.
CONSIDER:<br>*We are looking for selection bias which might compromise the generalisability of the findings:*<br>• *Was the sample representative of a defined population*<br>• *Was everybody included who should have been included*	
103. Were the measures accurately measured to reduce bias?	Yes. Objective metrics used which reduced bias. Accuracy, precision and sensitivity.
CONSIDER:<br>*Look for measurement or classification bias:*<br>• *did they use subjective or objective measurements*<br>• *do the measurements truly reflect what you want them to (have they been validated)*	
104. Were the data collected in a way that addressed the research issue?	Yes. With ethical approval
CONSIDER:<br>• *if the setting for data collection was justified*	
• *if it is clear how data were collected (e.g., interview, questionnaire, chart review)*<br>• *if the researcher has justified the methods chosen*<br>• *if the researcher has made the methods explicit (e.g. for interview method, is there an indication of*	

how interviews were conducted?)	
105. Did the study have enough participants to minimise the play of chance?	Yes. 530
CONSIDER:<br>• *if the result is precise enough to make a decision*<br>• *if there is a power calculation. This will estimate how many subjects are needed to produce a reliable estimate of the measure(s) of interest.*	
106. How are the results presented and what is the main result?	Using performance metrics in tables and charts.<br>Accuracy, precision, Sensitivity of Implant lengths:<br>Accuracy: 76%<br>Precision: 64%<br>Sensitivity: 59%
CONSIDER:<br>• *if, for example, the results are presented as a proportion of people experiencing an outcome, such as risks, or as a measurement, such as mean or median differences, or as survival curves and hazards*<br>• *how large this size of result is and how meaningful it is*<br>• *how you would sum up the bottom-line result of the trial in one sentence*	
107. Was the data analysis sufficiently rigorous?	Yes.
CONSIDER:<br>• *if there is an in-depth description of the analysis process*<br>• *if sufficient data are presented to support the findings*	
108. Is there a clear statement of findings?	Yes. The study concludes that the CNN model was effective in placing implants in the ideal position.
CONSIDER:<br>• *if the findings are explicit*<br>• *if there is adequate discussion of the evidence both for and against the researchers' arguments*<br>• *if the researchers have discussed the credibility of their findings*	

• *if the findings are discussed in relation to the original research questions*	
109. Can the results be applied to the local population?	Can’t tell.<br>Details of the demographic of the dataset are not at the forefront of the research, but still could provide useful information to draw firm conclusions
CONSIDER:<br>• *the subjects covered in the study could be sufficiently different from your population to cause concern.*<br>• *your local setting is likely to differ much from that of the study*	
110. How valuable is the research?	Yes. This research contributes to advancements in AI.
CONSIDER:<br>• *one descriptive/cross-sectional study rarely provides sufficiently robust evidence to recommend changes to clinical practice or within health policy decision making*<br>• *if the researcher discusses the contribution the study makes to existing knowledge (e.g., do they consider the findings in relation to current practice or policy, or relevant research-based literature?)*<br>• *if the researchers have discussed whether or how the findings can be transferred to other populations*	

**APPRAISAL SUMMARY**: *List key points from your critical appraisal that need to be considered when assessing the validity of the results and their usefulness in decision-making.*		
**Positive/Methodologically sound**	**Negative/Relatively poor methodology**	**Unknowns**
**Clearly defined method and results**<br>**Ethical approval**	**No validation from an expert**<br>**No time frame of dataset collection**	
